# Correlation between physical markers and psychiatric health in a Portuguese systemic lupus erythematosus cohort: The role of suffering in chronic autoimmune disease

**DOI:** 10.1371/journal.pone.0195579

**Published:** 2018-04-16

**Authors:** Margarida Figueiredo-Braga, Caleb Cornaby, Miguel Bernardes, Marta Figueiredo, Cristina Dos Santos Mesquita, Lúcia Costa, Brian D. Poole

**Affiliations:** 1 Medical Psychology Unit, Dep. Clinical Neurosciences and Mental Health, Faculty of Medicine, University of Porto, Porto, Portugal; 2 I3S Instituto de Investigação e Inovação em Saúde, Porto, Portugal; 3 Department of Microbiology and Molecular Biology, Brigham Young University, Provo, Utah, United States of America; 4 Rheumatology Department, Hospital of São João EPE, Porto, Portugal; 5 Departamento de Psicologia Aplicada (DPA), Universidade do Minho, Minho, Portugal; Universidade Federal do Rio de Janeiro, BRAZIL

## Abstract

**Background:**

Systemic lupus erythematosus (SLE) is a chronic autoimmune disease that affects a large number of people throughout the world. Anxiety, depression and fatigue are common symptoms of SLE that substantially contribute to decreased quality of life. This study investigates the interplay between physical and psychiatric manifestations of lupus. To this end, an SLE patient cohort was examined for correlations between clinical presentation, laboratory tests, and psychological indicators.

**Methods:**

Seventy-two lupus patients were evaluated for psychological status using a battery of instruments, including assessments for fatigue (CFS & FSS), depression (HADS), anxiety (HADS), overall health (SF-36 & PSQI) and intimate relationship satisfaction (RAS & CSI). Scores from these assessments were correlated with lupus clinical profiles and laboratory test values.

**Results:**

The prevalence of depression in the SLE patient cohort was 41.7%, as measured by the hospital depression and anxiety scale. The study identified that pain (p = 0.001), body mass index (p = 0.026), Chalder’s fatigue scale (p < 0.001), fatigue severity scale (p < 0.001), and anxiety (p = 0.001) are all positively correlated with depression in SLE patients. Total complement (CH50) (p = 0.032), and SF-36 physical and mental characteristic assessments are negatively correlated with depression. Longitudinal analysis indicated that the disease related complaint alopecia (p = 0.008) and relationship assessment scale scores (p = 0.004) may also be correlated to depression in SLE patients. Multivariant scrutiny of the clinical and psychosocial characteristics identified the fatigue severity scale (p = 0.026), SF-36 physical function (p = 0.040), physical role function (0.030), and mental health (p = 0.002) as the best indicators directly correlated with depression for the SLE cohort.

**Conclusion:**

These results reveal the influence of physical manifestations of lupus including fatigue, pain, body mass index and anxiety, as well as decreased physical and mental function, on depression. Fatigue is the strongest factor correlated with depression in SLE patients in the cohort. Both physical and social/psychological aspects likely contribute to the depression and anxiety in lupus.

## Introduction

Systemic Lupus Erythematosus (SLE) is an autoimmune disease that can affect a wide variety of organ systems. Symptoms vary and can include fever, arthritis, fatigue, weight loss, lymphadenopathy, a characteristic “butterfly rash”, renal disease and cytopenia, in a pleomorphic clinical presentation. Young women are predominantly affected by lupus, which brings significant costs into their personal, family and professional lives. Depression and fatigue are very common, early symptoms of lupus that are major contributors to diminished quality of life[1,2]. [3,4] These psychiatric symptoms are influenced by a variety of factors, and so understanding the causes, interactions, and consequences of depression and anxiety in lupus is a complicated and difficult problem. Factors that may influence depression and anxiety in lupus include culture, social and environmental factors, age, sex, disease activity and disease severity[5].

Both physical and psychological symptoms of lupus can affect the patient’s life. These could include such effects as weight and activity changes, fatigue and sleep disturbances. Moreover, a depressive disorder must be differentiated from more transient depressive symptoms[6]. Examination of the quality of depression, in addition to its prevalence, might yield more clues to the underlying mechanisms[7].

Due in large part to this complexity, the causes of depression in lupus are not clear. Biological mechanisms have been implicated because depression in lupus is associated with other CNS diseases, higher disease activity and more severe clinical manifestations[8,9]. Brain inflammation, interaction of auto-antibodies with antigens on neuronal cells membrane, and cytokine expression triggering neurotransmitter dysfunction have been regarded as possible causes of depression in SLE patients and in rodent SLE models[10–15].

Fatigue and pain are common symptoms in SLE, affecting up to 90% of the patients, who rate these symptoms as severe manifestations of the disease[16–18]. Neither clinical markers of disease activity nor medication (often used as a proxy indicator for disease severity) correlate well with levels of pain and fatigue[19]. Treatment of SLE may be substantially enhanced by better understanding fatigue and depression and their associations with immune activation[20].

The purpose of this study is to examine the diverse array of social, physical, and psychiatric factors that are interrelated with depression in lupus. We hypothesize that some of these features of lupus will correlate well with depression, and that these could aid clinicians in identifying and treating the depression and anxiety associated with lupus. We further hypothesize that some inflammatory or physical indicators of disease activity will be associated with psychological symptoms.

## Methods

### Patients

The studied population included 72 Caucasian lupus patients and 34 controls, recruited in northern Portugal. All SLE patients were previously diagnosed and followed at an outpatient unit. Diagnosis was established according to the American College of Rheumatology Criteria (ACR) guidelines and the duration of the disease was measured from the time when the patients first met the diagnostic criteria. The control group of patients with depression but without SLE was undergoing treatment at a private psychiatric clinic at the time of the study. The response rate for this group was 76%.

To account for inter-interviewer variation, psychiatric evaluation and psychometric markers were tested by one psychiatrist and one psychologist to establish the severity of depression. Exclusion criteria comprised history of substance abuse, personality disorders and or other major psychopathology than depression. Patients and controls were subsequently interviewed by phone by trained interviewers. The literature corroborates phone interviews as valid and precise tools for psychological data collection[21–23].

Participants’ socio-demographic data included age, educational level, employment status (active/non-active) and marital status (Table 1). Laboratory and SLE clinical evaluations were obtained for the SLE patients through the clinical records. Lab tests included leukocytes (10^9^/L), lymphocytes (percentage), neutrophils (percentage), platelets (10^9^/L), erythrocyte sedimentation rate (mm/h), anti-dsDNA antibody titer (IU/ml), C3 level (g/L), C4 level (g/L), CH50 level (U/ml), and C-reactive protein level (mg/dl). Other clinical symptomology also recorded from patients included cutaneous manifestations, photosensitivity, foot and mouth ulcers, arthritis, alopecia, headaches, kidney disease, neurological symptoms, pulmonary disease, musculoskeletal pathology, history of hypertension, and acute confusion syndrome (ACS). Smoking and alcohol consumption were also recorded.

**Table 1:** Sociodemographic Characteristics of the patient cohort.

*Characteristics*	All Subjects	SLE Subjects	SLE, non-depressed (HADS Depression ≤ 7)	SLE, depressed (HADS Depression ≥ 8)	SLE, non-depressed to SLE, depressed	Depressed	SLE total to Depressed	SLE, non-depressed to Depressed	SLE, depressed to Depressed
	(N = 106)	(N = 72)	(N = 42)	(N = 30)	*p-value*	(N = 34)	*p-value*	*p-value*	*p-value*
Gender, no. (%)									
Female	103 (97)	72 (100)	42 (100)	30 (100)		31 (91)			
Age, mean ± SD	46.64 ± 11.49	44.31 ± 9.9	41.55 ± 8.51	48.17 ± 10.53	0.004 a	51.59 ± 13.12	0.006 d	< 0.001 d	0.259 a
Education (years), mean ± SD	8.80 ± 3.80	8.8 ± 3.6	9.8 ± 3.18	7.43 ± 3.73	0.005 a	8.79 ± 4.26	0.991 a	0.244 a	0.182 a
Education Level, no. (%)					0.006 b		0.142 b	0.083 b	0.870 b
Primary	38 (36)	26 (36)	8 (19)	18 (60)		11 (32)			
Middle School	24 (23)	16 (22.2)	12 (28.6)	4 (13.3)		8 (24)			
High School	25 (24)	20 (27.8)	15 (35.7)	5 (16.7)		5 (15)			
College	19 (17)	9 (12.5)	6 (14.3)	3 (10)		10 (29)			
Marriage Status, no. (%)					0.667 b		0.070 b	0.214 b	0.161 b
Unmarried	16 (15)	11 (15.3)	8 (19.0)	3 (10)		6 (18)			
Married	78 (74)	53 (73.6)	30 (71.4)	23 (76.7)		25 (74)			
Divorced	9 (8)	8 (11.1)	4 (9.5)	4 (13.3)		1 (3)			
Widowed	3 (3)	0 (0)	0 (0)	0 (0)		3 (9)			
Employment Status, no. (%)					0.088 b		0.300 b	1.000 b	0.074 b
Employed	59 (56)	28 (38.9)	20 (47.6)	8 (26.7)		17 (50)			

^a^Independent samples t-test;

^*b*^ Fisher’s exact chi-squared test;

^*d*^ Welch’s t-test

The study was submitted and approved by the Ethical Committee of the São João Hospital IRB (EPE) in accordance with the Declaration of Helsinki. All participants gave written informed consent.

### Psychosocial evaluation

Socio-demographic characterization included age, education measured as years of school, marital status and socio-economic class evaluation. Psychological evaluations were obtained through a battery of standardized instruments.

#### Fatigue Severity Scale (FSS)

The short form of the FSS allows evaluation of self-reported fatigue[24]. The Portuguese version includes nine items and is recommended as the instrument of choice for research purposes in studies involving patients diagnosed with SLE[5].

The FSS demonstrates good psychometric properties (Cronbach’s α = 0.89 and test-retest reliability 0.84). A final score is obtained from the mean of all scored items, with higher scores revealing higher severity of fatigue. Presence of clinical levels of fatigue was defined by a FSS score >3. The scale has proved to be sensitive to change and reliable for telephone interviewing.

#### Hospital Anxiety and Depression Scale (HADS)

The Hospital Anxiety and Depression Scale (HADS) is a self-rating scale with good psychometric properties (Cronbach’s alpha coefficients of 0.94), designed to measure anxiety and depression in physically ill individuals[25]. Translated and adapted for Portugal[26,27], it is subdivided in two subscales of 7 items that measure independently anxiety and depression. The partial result of each scale varies between 0 and 21. Scores ranging from 8 to 10 are considered mild, from 11 to 14 moderate and 15 to 21 severe[28] and the authors suggest 8 as the cutoff point, considering values below as indicating the absence of anxiety and depression[25]. It is important to note that the scale is indicative of depressive symptoms in the last week, and not necessarily clinical depression.

#### Pittsburgh Sleep Quality Index (PSQI)

This instrument presents good psychometric properties, with high reliability (Cronbach’s alpha = 0.83) and validity. The seven components evaluated—sleep latency, sleep disturbances, sleep duration, sleep quality, sleep efficiency, use of sleep medications and daytime dysfunction allow the gathering of a global score varying from 0 to 21[29,30]. The PSQI is reliable for sleep quality assessment in telephone interviews and permits the identification of poor sleepers (score > 5)[31,32].

#### Chalder Fatigue Scale (CFS)

The Chalder Fatigue Scale[33], is an instrument with 11 items that evaluates the extent and severity of mental and physical fatigue using a five-point scale. Higher scores indicate higher fatigue.

#### Medical outcomes study questionnaire short form 36 health survey (SF-36)

The SF-36[34], Portuguese version[26], is a 36 item questionnaire that measures functional health and well-being in eight domains: physical functioning, role-physical, bodily pain, general health, vitality, social functioning, role-emotional, mental health and reported health transition. The instrument allows a score for each domain, as well as a global score. Higher scores indicate better health.

#### Couples Satisfaction Index (CSI)

The CSI[35] (Portuguese experimental version from Barbosa & Figueiredo-Braga, 2014) is a 32 item questionnaire that measures couple satisfaction in the relationship. Higher scores indicate greater satisfaction.

#### Relationship Assessment Scale (RAS)

The RAS[36] (Portuguese experimental version from Mesquita, Barbosa, & Figueiredo-Braga, 2014) is a 7 item instrument, with a five-point scale that measures general satisfaction with the relationship.

### Statistical analysis

Differences in the demographic, clinical, and psychological variables between the total SLE subjects, SLE non-depressed (LN), SLE depressed (LD), and depressed control (DC) subjects were determined using the independent t-test, Fisher’s exact chi-squared, Mann-Whitney U, Wilcoxon rank sum, or Welch’s tests when appropriate. Fisher’s exact chi-squared was used in place of the standard chi-squared test, which would typically be utilized, due to the smaller sample size of the groups compared. The statistical test used for the comparisons are indicated in the table legends. Multivariant analysis was performed using a generalized linear model. The best fit model was determined using the model with the appropriate number of variables (less than or equal to 8 variables) and the highest pseudo R^2^ value with the lowest approximate Akaike information criterion (AICc) value. In the multivariate analysis, the HADS depression score for all SLE subjects was used as the dependent variable and all candidate models included the confounding variables of age, education in years (socioeconomic status indicator), and body mass index. Statistical analysis was performed using the statistical software R and SPSS (IBM). An alpha value less than or equal to 0.05 was considered significant.

## Results

The systemic lupus erythematosus (SLE) patient cohort consisted of 72 females with a mean age of 44.31 years. 41.7% of SLE patients demonstrated pathological HADS depression scores, indicating current depressive symptoms. This prevalence, while high, is not uncommonly so for SLE populations[37,38].

A cohort of 34 physically healthy people who were diagnosed and being treated for depression acted as a control group (Hereafter referred to as DC, depressed control). This group was slightly older than the lupus cohort with a mean age of 57.69 years. For several analyses the SLE cohort was divided into two groups, those who demonstrated normal HADS depression scores (HADS < 8, n = 42, referred to as LN, Lupus non-depressed) and those who recorded above normal HADS depression scores (HADS ≥ 8 n = 30, referred to as LD, lupus depressed). To ensure that the DC group was comparable with the LD group, despite the selection of the lupus depression group by HADS score, the depressed non-lupus group was also stratified by depression, and only the depressed patients with HADS scores of ≥8 were compared to the lupus depression group. This selection still significantly demonstrated the same patterns that were observed using the entire depressed patient cohort.

The LD group displayed fewer years of formal education compared to the LN group (p = 0.005). When years of education are compared between LN patients and the DC group, the DC group has significantly fewer (p = 0.018), but there is no significant difference between the LD patients and DC patients (p = 0.871) (Table 1). Aside from years of education, the SLE cohort sub groups and the depressed patient control cohort share similar socioeconomic indicators such as marital and employment status.

### Clinical manifestations

The laboratory data were similar between the LD and LN groups save for the total complement (CH50) levels. These levels are significantly lower on average in the LD patients, who had a mean CH50 level of 9.81 (± 39.07) U/ml compared to the LN group that had a mean CH50 level of 45.88 (± 86.24) U/ml. This large difference is statistically significant (p = 0.032) (Table 2).

**Table 2:** SLE study cohort clinical characteristics.

*Characteristics*	SLE Subjects	SLE, non-depressed (HADS Depression < 8)	SLE, depressed (HADS Depression ≥ 8)	SLE, non-depressed to SLE, depressed	
	(N = 72)	(N = 42)	(N = 30)	*Odds Ratio (95% CI)*	*p-value*
Disease Duration	15.11 ± 8.47	14.57 ± 7.27	15.87 ± 9.99		0.526 a
Body Mass Index	24.55 ± 5.64	23.16 ± 3.63	26.49 ± 7.24		0.026 d
Pain Score, median	5.5	4	7.5		0.001 c
***Laboratory Results***					
Leukocytes (10^9^/L)	6.77 ± 2.15	6.88 ± 2.36	6.62 ± 1.85		0.514 a
Lymphocytes (%)	28.98 ± 8.11	29.58 ± 8.37	28.14 ± 7.81		0.443 a
Neutrophils (%)	58.78 ± 7.10	58.43 ± 7.53	59.27 ± 8.49		0.665 a
Platelets (10^9^/L)	228.64 ± 82.33	217.06 ± 90.42	244.62 ± 67.92		0.172 a
Sedimentation Velocity (mm/h)	22.67 ± 20.04	22.84 ± 21.49	22.43 ± 18.25		0.935 a
Anti-dsDNA (IU/ml)	99.89 ± 111.73	99.63 ± 116.53	100.30 ± 106.28		0.983 a
C3 (g/L)	44.23 ± 55.19	42.11 ± 53.4	47.17 ± 58.38		0.710 a
C4 (g/L)	8.67 ± 11.36	8.49 ± 10.71	8.9 ± 12.38		0.881 a
CH50 (U/ml)	32.05 ± 73.63	45.88 ± 86.24	9.81 ± 39.07		0.032 d
C-Reactive Protein (mg/dl)	0.43 ± 0.58	0.37 ± 0.6	0.49 ± 0.53		0.453 a
***Clinical Symptomology***					
Cutaneous Manifestations(%)	37 (51.4)	18 (42.9)	19 (63.8)	2.30 (0.88–6.03)	0.100 b
Photosensitivity, no. (%)	65 (90.3)	36 (85.7)	29 (96.7)	4.83 (0.55–42.45)	0.227 b
Foot & Mouth Ulcers, no. (%)	28 (38.9)	14 (33.3)	14 (46.7)	1.75 (0.67–4.58)	0.328 b
Arthritis, no. (%)	60 (83.3)	32 (76.2)	28 (93.3)	1.89 (0.34–10.48)	0.063 b
Alopecia, no. (%)	48 (66.7)	29 (69)	19 (63.3)	0.77 (0.29–2.08)	0.623 b
Headaches, no. (%)	40 (55.6)	17 (40.5)	23 (76.7)	4.83 (1.70–13.76)	0.004 b
Kidney Disease, no (%)	39 (54.2)	22 (52.4)	17 (56.7)	1.19 (0.46–3.05)	0.812 b
Neurological Symptoms (%)	19 (26.4)	9 (21.4)	10 (33.3)	1.83 (0.63–5.28)	0.289 b
Smoke, no. (%)	13 (18.1)	9 (21.4)	4 (13.3)	0.56 (0.16–2.04)	0.537 b
Alcohol, no. (%)	6 (8.3)	3 (7.1)	3 (10)	1.44 (0.27–7.70)	0.688 b
Neuropsychiatric pathology (%)	19 (26.4)	9 (21.4)	10 (33.3)	1.83 (0.63–5.28)	0.298 b
Pulmonary disease, no. (%)	19 (26.4)	10 (23.8)	9 (30)	1.37 (0.48–3.94)	0.596 b
Musculoskeletal Pathology (%)	11 (15.3)	6 (14.3)	5 (16.7)	1.2 (0.33–4.37)	1.000 b
Physically Active, no. (%)	24 (33.3)	17 (40.5)	7 (23.3)	0.45 (0.16–1.27)	0.204 b
Thyroid Pathology, no. (%)	5 (6.9)	3 (7.1)	2 (6.7)	0.93 (0.15–5.93)	1.000 b
History of Hypertension (%)	17 (23.6)	9 (21.4)	8 (26.7)	1.33 (0.45–3.98)	1.000 b
Acute Confusion Syndrome, (%)	3 (4.2)	2 (4.8)	1 (3.3)	0.69 (0.06–7.97)	1.000 b

^a^ Independent samples t-test;

^*b*^ Fisher’s exact chi-squared test;

^*c*^ Mann-Whitney U test;

^*d*^ Welch’s t-test

**Fig 1:**
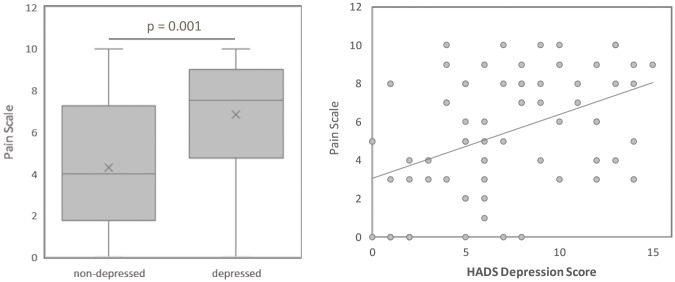
Pain and active depression are linked in lupus patients. **A**. Lupus patients exhibiting depressive symptoms report higher pain than patients without depressive symptoms. Pain was self-reported on a 1–10 scale. (p = 0.001). B. Pain and depressive symptoms are significantly positively correlated in lupus patients (p<0.0001). N = 72.

The LD patients reported higher pain scores than the LN subjects (p = 0.001). The LD group recorded a median pain score of 7.5, compared to 4 for the LN group. As HADS depression scores increase, the pain scores also increase, showing significant correlation (Fig 1, p<0.001). Along with the increase in pain, the LD patients further reported a higher frequency of headaches (p = 0.004) with 76.7% compared to 40.5% of the LN patients (Table 2).

An increase in body mass index (BMI) was observed in the LD cohort compared to the LN group (p = 0.026). LN subjects had a mean BMI of 23.16 (± 3.63) compared to 26.49 (± 7.24) in the LD group (Table 2). The DC group had a mean BMI of 25.41 (± 4.78). Interestingly, both the LD and DC groups have significantly higher BMIs than the LN group (p = 0.025). There was a higher percentage of subjects from the LD group that regularly consumed alcoholic beverages compared to either SLE group, with 32% compared 10% and 7.1%, for the LD and LN subjects respectively (p = 0.007 and p = 0.038).

### Psychosocial function

Both psychological tools used to assess fatigue severity demonstrated significantly different levels of fatigue between the LD and the LN patients (Table 3). Lower scores on the CFS indicate less fatigue, while higher scores indicate more fatigue. The LN subjects had less physical, mental, and overall fatigue than the LD patients (p = 0.001, p = 0.001, p < 0.001, respectively). The depression-only group CFS scores are not statistically different from either group.

**Table 3:** SLE patient psychological characteristics.

*Characteristics*	SLE Subjects	SLE, non-depressed (HADS Depression ≤ 7)	SLE, depressed (HADS Depression ≥ 8)	SLE, non-depressed to SLE, depressed	
	(N = 72)	(N = 42)	(N = 30)	*Odds Ratio (95% CI)*	*p-value*
**Chalder Fatigue Scale, median**	16	14	18		< 0.001 c
Physical Score	10	9	12		0.001 c
Mental Score	5	5	7		0.001 c
**SF-36 Scores**					
Physical Function, Median	22	24	17.5		< 0.001 c
physical Role Function, Median	11.5	14	9.5		0.001 c
Emotional Role Function, Median	9	11	7.5		< 0.001 c
General Health, mean ± SD	12.34 ± 3.27	13.32 ± 3.44	10.97 ± 2.46		0.002 a
Change of Health, Median	3	3	4		0.001 c
Social Role Function, Median	7	8	6		< 0.001 c
Bodily Pain	6.32 ± 2.86	7.43 ± 2.93	4.75 ± 1.87		< 0.001 d
Vitality, Median	10	11	7.5		< 0.001 c
Mental Health, Median	14.5	16.5	13		< 0.001 c
**HADS Anxiety**	9.31 ± 3.86	8.05 ± 3.75	11.07 ± 3.32		0.001 a
**HADS Anxiety Groups, no. (%)**					0.001 b
normal (HADS ≤ 7)	23 (31.9)	20 (47.6)	3 (10)	0.12 (0.03–0.47)	
mild to severe (HADS ≥ 8)	49 (68.1)	22 (52.4)	27 (90)	8.18 (2.15–31.18)	
**Pittsburg Sleep Quality Index**	8	8	9		0.076 c
**Pittsburg Sleep Quality Index groups (%)**					1.000 b
normal (PSQI ≤ 5)	10 (13.9)	6 (14.3)	4 (13.3)	0.92 (0.24–3.60)	
poor (PSQI ≥ 6)	62 (86.1)	36 (85.7)	26 (86.7)	1.08 (0.28–4.23)	
**Couple Satisfaction Index**	1479.83 ± 668.26	1551.85 ± 680.78	1374.35 ± 647.07		0.282 a
**Relationship Assessment Scale**	25.45 ± 4.19	25.48 ± 3.95	25.40 ± 4.56		0.869 c
**Fatigue Severity Scale**	4.73 ± 1.55	4.12 ± 1.65	5.59 ± 0.85		< 0.001 a

^a^ Independent samples t-test;

^*b*^ Fisher’s exact chi-squared test;

^*c*^ Mann-Whitney U test;

^*d*^ Welch’s t-test

HADS: Hospital Anxiety and Depression Scale; PSQI: Pittsburg Sleep Quality Index

The second fatigue assessment, the fatigue severity scale (FSS), also found statistical differences in fatigue between the LD and LN groups (p < 0.001). The LN group demonstrated a mean FSS score of 4.12 (± 1.65) compared to the LD group (5.59 ± 0.85). Interestingly, the Lupus patients as a whole had significantly higher levels of fatigue compared to the DC group (p = 0.002). The LD group also had significantly higher fatigue than the DC group (P<0.0001), but the LN group did not (p < 0.0001) (Table 4).

**Table 4:** Study cohort psychological characteristics.

*Characteristics*	SLE Subjects	SLE, non-depressed (HADS Depression ≤ 7)	SLE, depressed (HADS Depression ≥ 8)	SLE, non-depressed to SLE, depressed		Depressed	SLE Subjects to Depressed		SLE, non-depressed to Depressed		SLE, depressed to Depressed	
	(N = 72)	(N = 42)	(N = 30)	*Odds Ratio (95% CI)*	*p-value*	(N = 34)	*Odds Ratio (95% CI)*	*p-value*	*Odds Ratio (95% CI)*	*p-value*	*Odds Ratio (95% CI)*	*p-value*
Disease Duration (years), mean ± SD	15.11 ± 8.47	14.57 ± 7.27	15.87 ± 9.99		0.526 a	N/A		*N/A*		*N/A*		*N/A*
BMI, mean ± SD	24.55 ± 5.64	23.16 ± 3.63	26.49 ± 7.24		0.026 d	25.41 ± 4.78		0.443 a		0.023 a		0.491 d
Pain Score, median	5.5	4	7.5		0.001 c	5		0.333 c		0.017 c		0.257 c
*Smoking—no*. *(%)*					0.537 b			0.136 b		0.098 b		0.407 b
Yes	13 (18.1)	9 (21.4)	4 (13.3)	0.56 (0.16–2.04)		2 (6)						
*Alcohol—no*. *(%)*					0.688 b			0.003 b		0.007 b		0.038 b
Yes	6 (8.3)	3 (7.1)	3 (10)	1.44 (0.27–7.70)		11 (32)						
*Physically Actives—no*. *(%)*					0.204 b			0.291 b		0.817 b		0.114 b
Yes	24 (33.3)	17 (40.5)	7 (23.3)	0.45 (0.16–1.27)		15 (44)						
HADS Anxiety, mean ± SD	9.31 ± 3.86	8.05 ± 3.75	11.07 ± 3.32		0.001 a	9.15 ± 4.26		0.855 a		0.237 a		0.053 a
HADS Anxiety Groups, no. (%)					0.001 b			0.279 b		0.819 b		0.005 b
normal (HADS ≤ 7)	23 (31.9)	20 (47.6)	3 (10)	0.12 (0.03–0.47)		15 (44)	0.59 (0.26–1.38)		1.15 (0.46–2.86)		0.14 (0.04–0.55)	
mild to severe (HADS ≥ 8)	49 (68.1)	22 (52.4)	27 (90)	8.18 (2.15–31.18)		19 (56)	1.68 (0.73–3.89)		0.87 (0.35–2.15)		7.11 (1.80–28.00)	
Pittsburg Sleep Quality Index, median	8	8	9		0.076 c	7.5		0.075 c		0.360 c		0.018 c
Pittsburg Sleep Quality Index groups, no. (%)					1.000 b			0.067 b		0.157 b		0.142 b
normal (PSQI ≤ 5)	10 (13.9)	6 (14.3)	4 (13.3)	0.92 (0.24–3.60)		10 (29)	0.39 (0.14–1.05)		0.40 (0.13–1.25)		0.37 (0.10–1.34)	
poor (PSQI ≥ 6)	62 (86.1)	36 (85.7)	26 (86.7)	1.08 (0.28–4.23)		24 (71)	2.58 (0.95–6.99)		2.5 (0.80–7.79)		2.71 (0.75–9.79)	
Relationship Assessment Scale, mean ± SD	25.45 ± 4.19	25.48 ± 3.95	25.40 ± 4.56		0.869 c	21.32 ± 6.44		0.009 d		0.014 d		0.028 a
Fatigue Severity Scale, mean ± SD	4.73 ± 1.55	4.12 ± 1.65	5.59 ± 0.85		< 0.001 a	3.72 ± 1.63		0.002 a		0.224 a		< 0.001 d

^a^ Independent samples t-test;

^*b*^ Fisher’s exact chi-squared test;

^*c*^ Mann-Whitney U test;

^*d*^ Welch’s t-test

To assess the patients’ perception of their general health, the SF-36 assessment was used. This psychological tool yields eight sub scores, each of which was different between the LD and LN subjects. The SF-36 assessment gives a higher score for patients that feel healthier in that sub category, with lower scores indicating lower health. The LN cohort reported a median physical function score of 24, and a median physical role function score of 14. The LD patients demonstrated significantly lower physical function and physical role function scores, with median scores of 17.5 and 9.5 respectively (p < 0.001 and p = 0.001, respectively) (Table 3). The LN patients exhibited a mean general health score of 13.32 ± 3.44 and a median health transition score of 3, which was significantly higher than the LD, (general health 10.97 ± 2.46, health transition 4), (p = 0.002 and p = 0.001, respectively). The final sub score for physical health, bodily pain, in the LN cohort was had a mean of 7.43± 2.93, while the LD group had a mean of 4.75 ± 1.87. This difference in scores indicates a significantly higher level of perceived pain in the SLE depressed subjects (p < 0.001) (Table 3).

The mental health assessment portion of the SF-36 consists of the variable sub scores including vitality, social role function, emotional role function, and mental health. LN patients exhibited higher vitality and social role function scores (p < 0.001 and p < 0.001, respectively) with a median score of 11 and 8 respectively, than the LD patients, which had a median score of 7.5 and 6, respectively. Emotional health assessed by the SF-36 recorded that the LN subjects had a median score of 11, while the LD patients had a lower median score of 7.5 (p < 0.001). The final sub score calculated for the LN cohort, mental health, had a median score of 16.5 and the LD patients had a median score of 13 (p < 0.001).

The LD patients were highly anxious, with 90% demonstrating heightened anxiety scores compared to the LN subjects, who had 52.4% with HADS anxiety scores indicating above normal anxiety (p = 0.001). The DC cohort exhibited similar anxiety scores to the SLE non-depressed control with 56% of the subjects having higher than normal anxiety scores. These values are significantly lower than those observed in the SLE depressed patients (p < 0.001).

### Longitudinal impact

**Fig 2:**
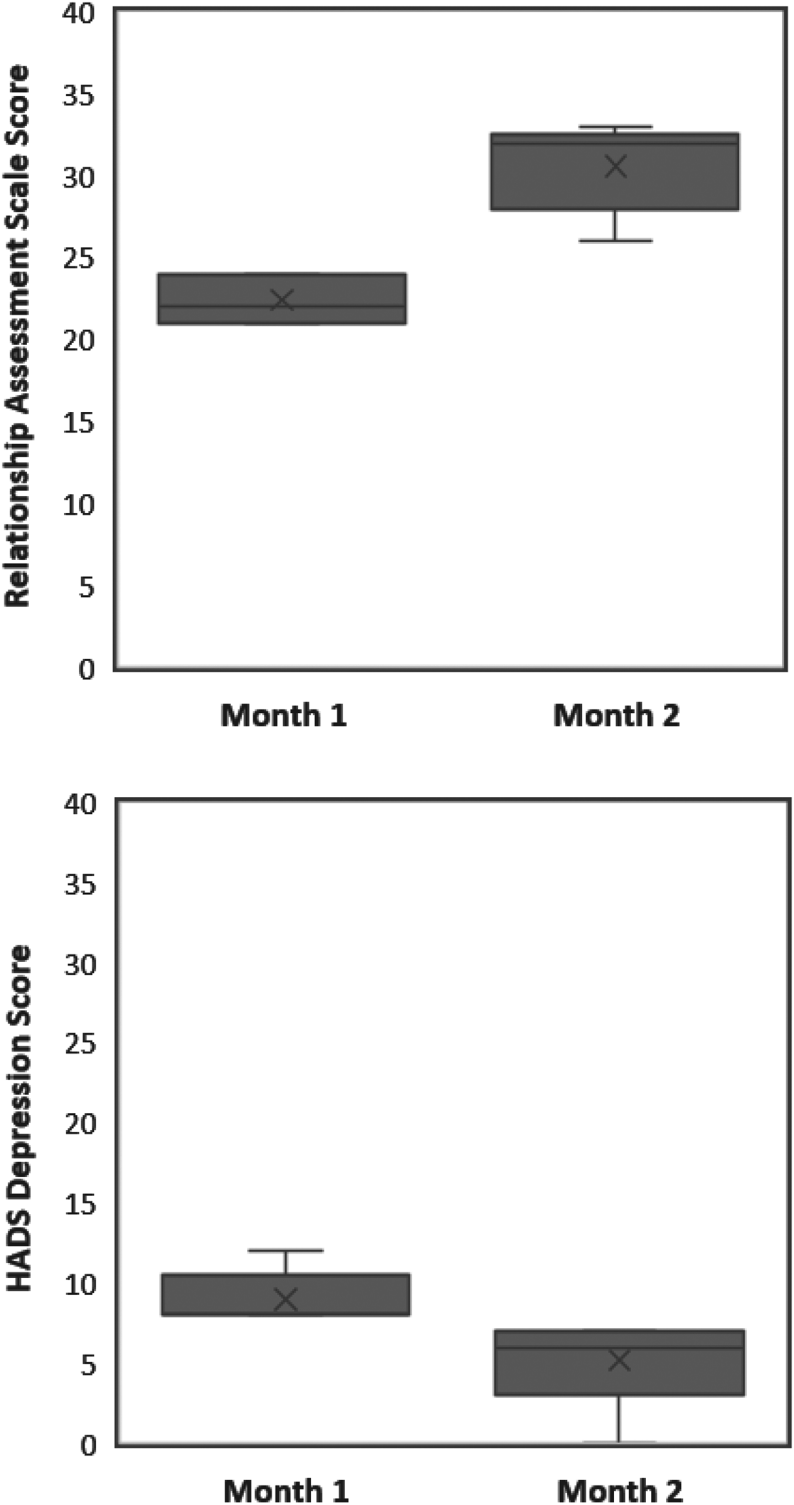
Lupus patients whose depression improves also have improved relationship satisfaction. Five lupus patients who had depressive symptoms at the onset of the study improved their HADS depression scores to the point where they were considered non-depressed. These patients showed concomitant increases in relationship satisfaction, as measured by the Relationship Assessment Scale. P = 0.004, N- = 5.

Approximately one month after the initial clinical and psychosocial assessment, patients from the SLE cohort were reassessed. Of thirty SLE patients that exhibited pathological HADS depression scores, five of them had normal HADS depression scores during this second assessment. While five patients compose a rather small group, these differences might spark further studies. In the first assessment, every one of these patients exhibited alopecia as a disease related complaint, however, in the second assessment, all five no longer had alopecia (p = 0.008). A further difference identified was the strong increase in relationship assessment scale (RAS) scores (p = 0.004). The first assessment revealed a mean relationship assessment scale score of 22.40 ± 1.52, while the second assessment recorded a mean relationship assessment scale score of 30.60 ± 2.79 (Fig 2).

### Multivariate analysis

To determine what variables might play a role as the best indicators of increased HADS depression scores in the LD relative to the LN groups, we utilized generalized linear regression models for the multivariate analysis. A list of candidate models was compiled and compared to one another based on the number of variables included, pseudo R^2^, and AICc values. Each model included the confounding sociodemographic variables that were identified as significantly different between the LD and LN subjects, namely age, body mass index, and years of education. All other variables that were found to be significantly different between the LN and LD patients were included as possible variables in the multivariant analysis. The only significant variable not included as a candidate variable was HADS anxiety scores. This variable was omitted due to the close correlation with the HADS depression score and because they are derived from the same assessment. The best fit model identifies the best indicators of depression for this SLE cohort. Of the variables included in the model, several were considered significant. These include three SF-36 scores, physical function (p = 0.040), physical role function (p = 0.030), and mental health (p = 0.002), as well as fatigue severity scale scores (p = 0.029). Of these, the latter is likely the most sensitive to changes in HADS depression scores as indicated by the odds ratio of 1.11 (1.01–1.22), which is slightly higher than other significant variables (Table 5).

**Table 5:** Multivariate model for SLE cohort.

*Variables*	*Odds Ratio (95% CI)*	*p-value*
Age (years)	1.01 (0.99–1.02)	0.277
Education (years)	0.99 (0.96–1.02)	0.451
Body Mass Index	1.01 (1.00–1.02)	0.123
SF-36 Score		
*Physical Function*	0.97 (0.95–1.00)	0.040
*Physical Role Function*	1.04 (1.00–1.07)	0.030
*Vitality*	0.97 (0.93–1.01)	0.180
*Mental Health*	0.96 (0.93–0.98)	0.002
Fatigue Severity Scale	1.11 (1.01–1.22)	0.029
Model Fit Summary	*pseudo R2*	*AICc*
	0.787	367.720

## Discussion

The overwhelming finding of this study is that lupus patients with depression are experiencing extreme suffering. They have a higher number of depressive symptoms even than patients for whom depression is the primary diagnosis. They are also more prone to experience anxiety. In addition to being more depressed and anxious than primary depression patients, they suffer more physical symptoms than lupus patients without depression. These depressed lupus patients are living in the worst of both worlds.

Of the variables examined in this study, fatigue was the strongest indicator for depression in the lupus patients. This was observed in the univariate analysis, in the Chalder Fatigue Scale and Fatigue Severity Scale, and the multivariate analysis, making the measurement of fatigue an excellent indicator of depression in SLE patients (Table 3). Fatigue has previously been correlated to an increased risk for depression among lupus patients[1,39–41], and this study strengthens these associations.

Interestingly, considering the strong association between fatigue and depression, the LD and LN cohorts did not have a significant difference in sleep quality. While the prevalence of poor sleepers is equal to or higher than that observed in the general population, sleep quality does not appear to be correlated with depression, indicating that a poor sleep quality is most likely not causing depressive symptoms, and perhaps is not one of the significant factors contributing to such drastically high levels of fatigue among lupus patients[42]. However, overlying symptoms may be masking the effects of sleep quality in this population.

Fatigue has been found to correlate with increased body mass index in SLE patients[41]. This study supports this work (Table 2). Increased body mass index scores correlated with increased levels of depression amongst in SLE cohort. These results emphasize the importance of assisting patients alleviate chronic fatigue, as fatigue seems to be associated with a variety of psychosocial and clinical aspects of SLE. The findings from the fatigue assessments demonstrate that the depressed lupus cohort suffers from both physical and mental fatigue. The need for continued research to better understand how to best treat both types of patient fatigue is therefore potentially one of the most important ways to promote lupus patient quality of life.

The SF-36 psychosocial assessments were also excellent indicators of depression for the lupus cohort. All the physical and mental categories were negatively correlated with depression (Table 3). Two of the SF-36 physical components, physical and physical role function, were directly correlated with depression scores in the multivariate analysis. These findings support multiple studies that indicate SF-36 scores are negatively correlated with depression in SLE subjects[1,43]. It has been noted that patients experiencing symptoms of depression may understate their physical and mental functioning, leading to lower scores across the board, such as we observed. However, even if this is the case, these lower scores are still indicative of depression and could be useful in identifying patients in special need of psychiatric care.

Interestingly, the DC group show significantly higher scores in these three categories and most of the other SF-36 categories when compared to the SLE depressed subjects. These results suggest that the SLE depressed patients decreased physical and mental health is likely a result of SLE disease activity as opposed to depression. Another hypothesis could be the cumulative burden of the disability imposed by an autoimmune and a mental disorder severely diminishes quality of life.

Heightened pain correlated with increased depression in this study. This evidence confirms various studies demonstrating that increased pain is related to greater risk for depression in SLE patients[44,45]. Also of interest is the higher frequency of headaches observed in the LD population compared to the LN patients. A final interesting clinical characteristic is the decreased levels of total complement (CH50) observed in the depressed patients. This supports previously observed changes in complement levels in patients with neuropsychiatric lupus and animal models[44,46,47]. Low complement is a marker of inflammation and disease activity in lupus, and suggests that heightened immune dysfunction and associated disease activity correlates with more depression. Interestingly, other commonly used indicators of disease activity, such as C-reactive protein levels or Sedimentation Rate, do not correlate with depression. These tests also failed to correlate with any lupus symptoms in this cohort.

The lupus patients were re-evaluated a month after their initial enrollment. Five patients who were exhibiting depressive symptoms upon the first evaluation were no longer at the second contact. All of these patients had alopecia on their first contact but it was gone by their second assessment. They also rated their relationships as more satisfying. Increased satisfaction with a close relationship in SLE patients is likely a protective factor resulting in decreased depressive symptoms. Supporting and gratifying relationships are known to alleviate depression associated with other chronic and debilitating diseases[48]. However, with the small sample size and the nature of the experiment, no conclusive directionality can be imposed upon the data.

In the SLE cohort we observed a high prevalence (41.7%) of patients with active depressive symptoms. A still higher proportion of SLE subjects reported above average anxiety scores, 65.9%.

In summary, this cohort provides a comprehensive look into the relationship between depression and clinical and psychosocial variables in lupus. The findings highlight the importance of assisting patients with both physical and mental health care, and identify symptoms and markers that suggest care for depression may be likely. Beyond clinical and laboratory monitoring of immune dysfunction, attentive clinicians are aware of the value of more discrete symptoms as fatigue, pain, anxiety and depression. It has been demonstrated in past studies that treatment of the neuropsychological symptoms improves overall health, and appropriate treatment of physical symptoms assists in the improvement of psychological manifestations[44,49].

## Supporting information

S1 Dataset All data gathered for this study. (XLSX)
